# Age-related enhancement in visuomotor learning by a dual-task

**DOI:** 10.1038/s41598-022-09553-7

**Published:** 2022-04-05

**Authors:** Tony S. L. Wang, Miles Martinez, Elena K. Festa, William C. Heindel, Joo-Hyun Song

**Affiliations:** Department of Cognitive, Linguistic, and Psychological Sciences, Brown University, Providence, RI 02912 USA

**Keywords:** Psychology, Human behaviour

## Abstract

Many daily activities require performance of multiple tasks integrating cognitive and motor processes. While the fact that both processes go through deterioration and changes with aging has been generally accepted, not much is known about how aging interacts with stages of motor skill acquisition under a cognitively demanding situation. To address this question, we combined a visuomotor adaptation task with a secondary cognitive task. We made two primary findings beyond the expected age-related performance deterioration. First, while young adults showed classical dual-task cost in the early motor learning phase dominated by explicit processes, older adults instead strikingly displayed enhanced performance in the later stage, dominated by implicit processes. For older adults, the secondary task may have facilitated a shift to their relatively intact implicit learning processes that reduced reliance on their already-deficient explicit processes during visuomotor adaptation. Second, we demonstrated that consistently performing the secondary task in learning and re-learning phases can operate as an internal task-context and facilitate visuomotor memory retrieval later regardless of age groups. Therefore, our study demonstrated age-related similarities and differences in integrating concurrent cognitive load with motor skill acquisition which, may in turn, contributes to the understanding of a shift in balance across multiple systems.

## Introduction

With advancing age, we may notice ourselves having greater difficulty recalling a list of items to buy at the grocery store or walking a little more slowly than young adults. Healthy aging has, in fact, been associated with a decline across a variety of cognitive and motor functions, with older adults displaying particular difficulty compared to their younger counterparts when they attempt to perform a cognitive task while also performing a motor task in parallel^1–5^.

These age-related impairments in dual-task performance are likely due partly to the finding that motor control is more attentionally demanding for older than younger adults, requiring greater reliance on top-down cognitive resources to compensate for emerging sensory and motor deficits^6–12^. Indeed, older adults have been found to display greater utilization of cognitive resources than young adults for even relatively simple actions such as maintaining standing balance and walking, even though these simple actions have been thought to be implicit and automatic^8,13^. This increased need for attentional resources during motor tasks, along with additional age-related declines in visual attention capacity may lead to the disproportionate dual-task impairment seen in older adults compared to young adults^14^.

To date, however, the majority of studies examining age-related deficits in dual-task performance have focused on the effects of performing a stable secondary motor task (e.g., target tracking) on the performance of a primary cognitive task (e.g., visual search); only a few including the current study have examined how aging influences the effect of performing a secondary cognitive task on the primary task of acquiring a new motor skill^15^. According to a representative model of motor skill acquisition^16^, motor skill learning progresses through three interrelated stages that involve varying degrees of cognitive resources and control. First, learners must use their attentional resources to break down the desired skill into discrete components in the *cognitive stage*. This stage involves creating a mental picture of the skill, which helps to facilitate an understanding of how these parts come together to form the correct execution of the desired movement. Performance at this stage might be characterized as more controlled information processing for movement. Next, the *associative stage* involves repeated practice and feedback to connect the parts smoothly. Finally, the *autonomous stage* involves the continued development of the learned skill to become habitual and automatic. Individuals at this stage rely on processes that require little or no conscious attention. Furthermore, previous studies have demonstrated that dissociated explicit and implicit processes contribute to the visuomotor adaptation task and these processes are also map onto cognitively driven fast learning processes that dominate in the early learning stage and autonomous slow learning processes that dominate in the late learning stage, respectively^17–19^. Thus, here, we conceived that while early stages of skill acquisition learning are thought to depend primarily on explicit learning processes, later stages of skill learning depend primarily on implicit learning processes.

**Figure 1:**
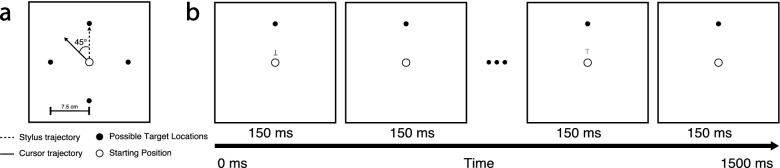
Task schematics. (**a**) Visuomotor adaptation task*.* The filled circles indicate possible target locations, and the open circle indicates the starting base. Reach targets appeared one at a time and remained visible for the entire trial (1500 ms). In no-rotation trials, the cursor followed stylus motion normally, whereas in rotation trials, the cursor direction was rotated by 45° counter-clockwise from the reach trajectory. The dashed lines show the trajectory of the stylus and of the cursor on rotation trials. There were four phases of visuomotor adaptation: (1) baseline (40 no-rotation trials), (2) learning (160 rotation trials), (3) washout (80 no-rotation trials) and (4) re-learning (80 rotation trials with four target directions). (**b**) Secondary rapid serial visual presentation (RSVP) task. A sequence of five ‘T’s was generated from pseudorandom permutations of letter orientation (inverted or upright) and color (red, white, green, blue or yellow). The sequence was presented serially directly above the starting position, and each letter was displayed for 150 ms with a 150 ms inter-stimulus interval. A target was defined as green ‘T’s. Participants had to report at the end of each trial how many relevant targets (one, two, or three) were presented in that trial.

To investigate how healthy aging interacts with the different stages of visuomotor skill acquisition during the concurrent performance of a secondary cognitive task, we used a newly developed dual-task paradigm in which we combined a primary visuomotor adaptation task (Fig. 1a) with an attention-demanding secondary rapid serial visual presentation (RSVP) task (Fig. 1b)^20–23^. Visuomotor adaptation is a type of motor learning that has been used extensively to examine how humans represent and interact with their environment. Adaptation involves the modification of a well-learned sensorimotor transformation and reflects our ability to adapt our motor performance in response to environmental changes, such as walking up a flight of stairs while wearing new prescription glasses.

As with other motor skills, recent studies in younger adults have confirmed that attentional or explicit learning processes play a greater role in the early stages of visuomotor adaptation while automatic or implicit learning processes play a greater role in the later stage^5,24–26^. In a recent fMRI study, for example, Anguera et al.^5^ found that early but not late learning rate on a visuomotor adaptation task was correlated with individuals’ spatial working memory performance and that the neural correlates of earlier but not later stages of visuomotor adaptation overlapped with the same brain regions that were engaged during performance of the spatial working memory task. Thus, for young adults, the concurrent performance of a secondary cognitive task would be expected to selectively impact the more attentionally-demanding early stage of visuomotor adaptation rather than the more automatic later stage.

For older adults, however, the predictions are not as straightforward. On the one hand, previous studies have consistently found that healthy aging primarily affects the early, explicit stage of visuomotor adaptation, leaving later implicit adaptation learning relatively intact^4,27–31^. Moreover, unlike young adults, early visuomotor adaptation in older adults has not been correlated with spatial working memory performance. Older adults display little neural overlap in the regions activated during early adaptation and spatial working memory tasks^32^. Taken together, these findings suggest that the decrements in adaptation learning displayed by older adults are due at least in part to a failure of older adults to effectively engage in explicit spatial working memory processes during the early stage of learning. From this perspective, older adults would be predicted to show disproportionately greater dual-task decrements in visuomotor adaptation compared to young adults that is attributable to additional disruption of the early stage of learning (the *resource depletion hypothesis*)^15^. That is, the increased attentional demands associated with performing the secondary task would exacerbate the already deficient allocation of spatial working memory processes critical for the early stage adaptation, thereby producing greater visuomotor adaptation impairment in the dual-task than the single-task condition compared to younger adults.

There are, however, two alternative hypotheses that would predict enhanced visuomotor adaptation learning in older adults under dual-task compared to single-task conditions. First, several recent studies have found that, under certain circumstances, young adults can demonstrate enhanced motor learning under dual-task compared to single-task conditions^33,34^. This enhanced learning has been attributed to the concurrent engagement of similar cognitive processes shared by the primary and secondary tasks. That is, some secondary tasks may facilitate the engagement of the same neural systems that are also important for learning the primary motor task, and this engagement, in turn, facilitates learning of the motor task^33,34^. Given that the visuomotor adaptation deficit in older adults is characterized by a failure to effectively engage explicit spatial working memory processes during the early stage of learning, the concurrent engagement of spatial working memory by a secondary cognitive task may facilitate the utilization of these processes in support of visuomotor adaptation by older adults. Thus, a *concurrent engagement hypothesis* would predict that certain dual-task conditions could improve visuomotor adaptation learning in older adults through the selective enhancement of the early, explicit stage of learning.

Second, another recent study found that, in young adults, explicit and implicit learning components of visuomotor adaptation are differentially associated with individual differences in working memory capacity. Working memory capacity is positively associated with explicit learning but negatively associated with implicit learning^35^. These findings suggest that the explicit and implicit learning components may operate in a “push–pull” manner, with individuals with low working memory capacity compensating for the reduced contribution of explicit processes through an enhanced contribution of implicit adaptation processes, and individuals with high working memory capacity relying more heavily on the contribution of explicit processes. Given their age-related decrements in working memory^15,32^, the increased working memory demands elicited by the concurrent secondary task may serve to accelerate a shift in older adults to a greater reliance on their relatively intact implicit adaptation processes and a reduced reliance on their already-deficient explicit adaptation processes. Thus, a *compensatory hypothesis* would predict that in older adults, dual-task conditions could improve visuomotor adaptation learning through shifting to greater reliance on implicit processes that are particularly beneficial during the late stage of learning.

In the present study, we observed the expected overall deterioration of performance in older adults compared to young adults on both the visuomotor adaptation learning task and the secondary cognitive task. However, we found opposite effects of the secondary cognitive task on the primary adaptation learning task in the two age groups. In young adults, we demonstrated that performing the concurrent secondary task led to decreased visuomotor adaptation characterized by poorer performance in the early, explicit stage of learning. In contrast, we observed a striking benefit of the concurrent secondary task on visuomotor adaptation in older adults that was characterized specifically by enhanced performance in the later, implicit stage of learning. Thus, these findings support predictions of the *compensatory hypothesis* that the attention-demanding secondary task facilitated a shift in older adults to a greater contribution of their relatively intact implicit learning processes to support visuomotor adaptation.

In addition, to evaluate the competing hypotheses regarding the effect of dual-task conditions on the motor learning phase in older adults, we also examined the relation between the dual-task context and their later re-learning phase. In a series of recent studies with young adults using a similar dual-task paradigm^20–23^, we discovered that visuomotor adaptation learned under dual-task conditions was retrieved during a later recall test only when a similar secondary task was present at re-learning as well. When participants were tested at re-learning without the secondary task, their performance reverted to untrained levels as though the motor task had not been learned in the first place. Hence, this result suggests that the dual-task context acts as a vital context for motor learning and performance. Until now, this task-context effect has not been examined in older adults. Here, we observed that like young adults, older adults who acquired visuomotor adaptation with the secondary task in the learning phase performed better when the secondary task was also present in the re-learning phase (*consistent context*) compared to when the secondary task was not present during re-learning (*inconsistent context*).

Taken together, we found both similarities and differences between young and older adults when they attempt to learn a new motor skill under cognitive loads. While we confirmed that older adults displayed worse adaptation learning overall than young adults as expected, we made two noteworthy discoveries. First, while the secondary task primarily *impairs* the early explicit learning phase in young adults, the secondary task does not have a noticeable effect on this early phase in older adults and *facilitates* later implicit learning consistent with the *compensatory hypothesis*. Secondly, we demonstrated that, like young adults, the later retrieval of a previously learned visuomotor skill in older adults also depends on the task-context.

## Results

In both young and old groups, participants performed the visuomotor adaptation task (Fig. 1a) with or without the RSVP task (Fig. 1b) depending on the requirements for the groups and experimental phases, as indicated in Table1.

**Table 1:** The secondary (RSVP) task performed in each group throughout each visuomotor adaptation phase.

Group	Visuomotor adaptation phase				
	Baseline (40 trials)	Learning (160 trials)	Washout (80 trials)		Re-learning (80 trials)
Single-task	None	None	None		None
Dual-task	RSVP	RSVP	None	Consistent	RSVP
				Inconsistent	None

The labels in Table 1 indicate whether participants performed the RSVP during each of the visuomotor adaptation phases such as baseline, learning, washout, and re-learning. Note that the stream of ‘T’s appeared on every trial of all experimental phases, so the visual stimuli were the same across all participants. When they performed the RSVP task, they had to report at the end of each trial how many green ‘T’s (one, two, or three) were presented in that trial. Otherwise, they pressed a key in response to a visual cue at the end of each trial (e.g., “Press button 1”). For instance, the *dual-task* group performed the visuomotor adaptation task concurrently with the attention-demanding RSVP task during the learning phase, whereas the *single-task* group only performed the visuomotor adaptation task. In the dual-task group, we further categorized participants during the re-learning phase depending on whether they continued to perform the RSVP task (*context consistent*) or not (*context inconsistent*).

### Age-related decline in the attention task and visuomotor adaptation during the learning phase

We first examined performance accuracy in the secondary (RSVP) task in the dual-task groups to confirm that participants had effectively allocated their attention to the secondary task in the learning phase. We observed that the RSVP accuracy in both young, Mean (M) = 69% ± 2.3, t(27) = 15.95, p < 0.001, d = 3.01, and old adults, M = 58% ± 2.9, t(29) = 8.4, p < 0.001, d = 1.53, were significantly above chance (33.33%). It showed that all participants were properly engaged in the secondary task during visuomotor learning. As expected, we confirmed that older adults have impaired performance in the secondary task compared to younger adults, F(1, 56) = 9.19, p < 0.01, Mean squared error (MSE) = 2.0, η^2^ = 0.14.

**Figure 2:**
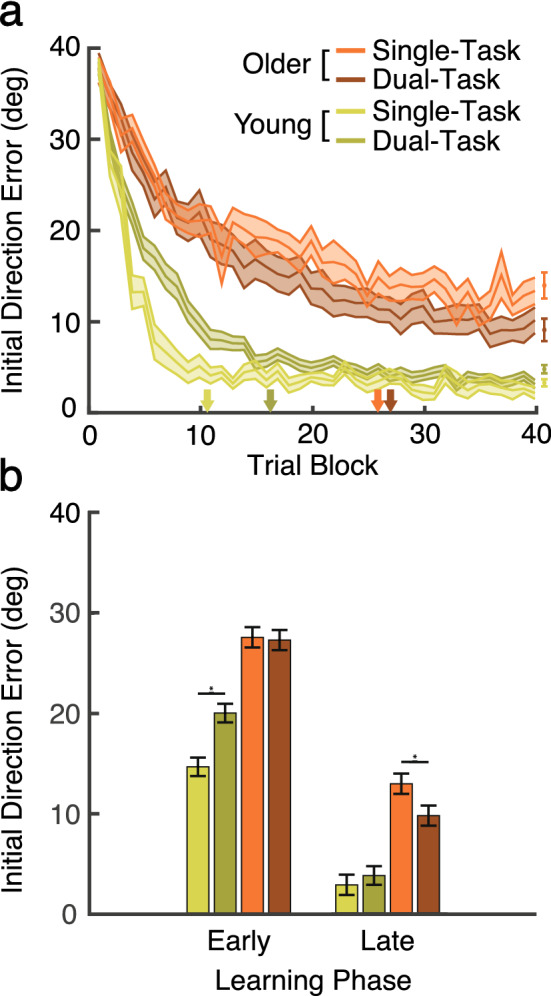
Visuomotor adaptation performance in the learning phase. We compare initial direction errors from young (green) and older (orange) adults across the single task (lighter color) and dual-task groups (darker color). (**a**) Initial direction error. The single dot data points on the far right (after the 40th trial block) indicate the estimated asymptotic reach value for each group. The arrows on the abscissa indicate the trial block at which each group reached asymptotic performance. The ribbons represent between-subjects standard error (SE). (**b**) Mean initial direction error in the early and late learning phases. Error bars indicate standard error (SE).

Then, we examined an overall age-related effect on visuomotor adaptation. Initial direction errors were reach errors at peak velocity (see “Data analysis” section in “Methods” section for details). Figure 2 shows initial direction errors of young (green) and older (orange) adults, who performed the single (lighter color) and the dual-task (darker color) separately as a function of trial block. Each trial block represents an average from four successive trials, in which one repetition of each target was presented (see “Data analysis” section in “Methods” section for details). As depicted in Fig. 2a, all age groups demonstrated an overall reduction of initial direction errors by the end of the learning phase. Furthermore, as expected, younger adults showed much better visuomotor adaptation in accord with previous studies investigating age-related differences in similar visuomotor learning paradigms^5,32^. We confirmed these observations with a three-way *age* (young vs. older adult) × *secondary task* (single vs. dual) × *trial block* (1st–40th trial block) ANOVA to confirm these observations: a significant main effect of age, F(1, 81) = 122.86, p < 0.01, η^2^ = 0.57, and trial blocks, F(14.18, 1176.99) = 235.95, p < 0.01, η^2^ = 0.74, MSE = 45.77. To sum up, both age groups learned the adaptation, but young adults produced smaller initial direction errors across the learning phase.

### Age-related benefits of the dual-task during the learning phase

Now, we focused on our primary interest of whether and how performing the simultaneous secondary RSVP task impacted visuomotor adaptation during the learning phase differently for the young and older adults. As shown in Fig. 2a, the secondary task modulates visuomotor adaptation differently depending on age and learning phases. Specifically, it appears that the secondary task *impairs* mostly the early learning phase (i.e., *cognitive* stage) in young adults (green), whereas the secondary task *facilitates* the later learning phase (i.e., *autonomous* stage) for older adults (orange). This observation was also confirmed by significant interaction effects of *age* × *secondary task* ANOVA, F(1, 83) = 7.02, p = 0.01, η^2^ = 0.8, and the three-way interaction of *age* × *secondary task* × *trial block*, F(14.18, 1176.99) = 2.05, p < 0.01, η^2^ = 0.02.

To further quantify age-related different processes during visuomotor learning, we analyzed mean initial direction error in the early (first 10 trial blocks) and late learning phase (final 10 trial blocks) for each age group. As illustrated in Fig. 2b, in the early phase, young adults (green bars) showed significantly larger initial direction errors in the dual-task (dark) than single-task group (lighter), demonstrating that the secondary task interferes with the cognitive stage of visuomotor adaptation, t(40) =  − 3.64, p < 0.01, d =  − 1.91, while older adults (orange bars) did not show a significant impairment by the dual-task (t < 1). As the learning progressed, we observed the opposite pattern in young and older adults in the late learning phase. Young adults in the dual-task group overcame their early-stage dual-task cost and reached the equivalent visuomotor adaptation level compared to the single-task group (t(40) =  − 1.65, p = 0.11). However, there is the possibility that the equivalent adaptation levels could be led by a floor effect. Regardless, the older adult participants showed lower initial direction errors in the dual-task than the single-task group, indicating the benefit from performing the simultaneous RSVP task in the late learning phase, t(43) = 2.02, p < 0.05, d = 0.64.

In sum, performing the secondary task appears to have different effects on visuomotor adaptation for young and older adults. Furthermore, the counter-intuitive benefit of the secondary task in the late learning phase for older adults was consistent with the *compensatory* hypothesis rather than the *concurrent engagement hypothesis*.

**Figure 3:**
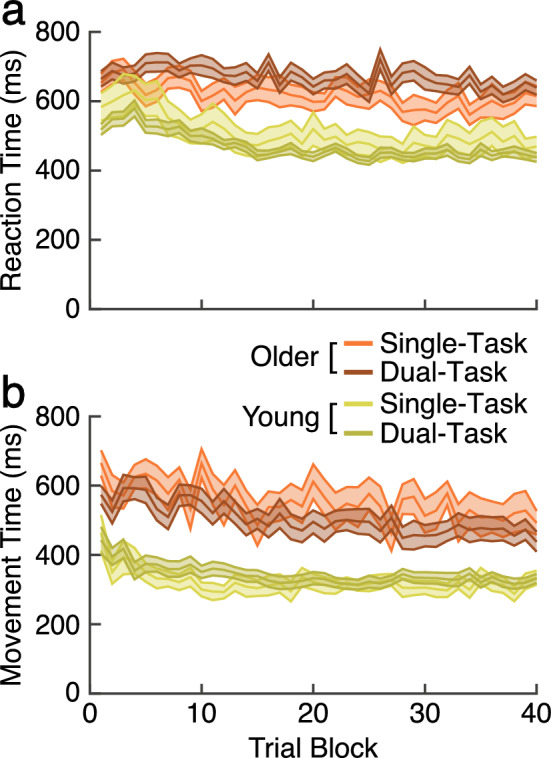
Reaction time (RT) and movement time (MT) in the learning phase. We compare performance in young (green) and older (orange) adults across the single task (lighter color) and dual-task groups (darker color). Ribbons indicate SE. (**a**) Reaction time. (**b**) Movement time.

We also analyzed reaction time and movement time to determine whether participants used a different reaching strategy in the dual-task procedure. For instance, participants may allocate extra cognitive resources to the RSVP task, causing delays in initiations of movement (RT) or slower movements (MT). In such cases, RT and/or MT would be longer in the dual-task groups. However, while not surprisingly younger participants were faster to initiate (RT) and move (MT) compared to older adult participants, within each age group, reaction time and movement time were equivalent across the single and dual-task groups, as shown in Fig. 3a,b, respectively.

Specifically, while young adults were faster to initiate movement compared to older adults, both age groups commonly reduced RT across trial blocks. The *age x secondary task x trial block* ANOVA confirmed these observation: significant main effects of age, F(1, 83) = 66.57, p < 0.01, η^2^ = 0.55, MSE = 0.28, and trial block, F(16.82, 1396.47) = 9.02, p < 0.01, η^2^ = 0.01, MSE = 0.02. However, the main effect of the secondary task was not significant (F < 1). Simple main effect analyses of each age group also confirmed that the mean RT is not different between the single and dual-task groups for either the young (F < 1) or older adult participants, F(1, 43) = 3.76, p = 0.06.

Similar pattern was also observed in MT: significant main effects of age, F(1, 83) = 63.9, p < 0.01, η^2^ = 0.44, MSE = 0.47 and block, F(16.84, 1398.08) = 6.9, p < 0.01, η^2^ = 0.08, MSE = 0.03. However, the effect of secondary task was not significant, F(1, 83) = 2.13, p = 0.15, η^2^ = 0.3. Simple main effect analysis of each group also showed that MT was not affected by the secondary task for both young (F < 1) and older adult, F(1, 43) = 1.53, p = 0.22. These observations in RT and MT are consistent with our previous studies that have used a similar dual-task paradigm^21–23,36^.

### Age independent task-context-consistency between learning and re-learning phases

So far, we have focused on how allocating attentional resources to a concurrent secondary task modulates age-related performance in visuomotor adaptation during the learning phase. Here, we examined how performing the secondary task affects memory formation and re-learning. According to our recent work in young adults^20–23^, when participants were tested later during the re-learning phase, a visuomotor adaptation learned under the dual-task was remembered only when a similar secondary task was present, while when they were tested without the secondary task, their performance reverted to untrained levels as though the motor task had not been learned in the first place. Hence, this result, in which the level of performance decreases when more attentional resources are available, suggests that the dual-task context, or the lack thereof, acts as a vital context for learning^20–23^.

We analyzed those who performed the dual-task in the learning phase to determine whether the consistent dual-task context is helpful for motor memory retrieval in the re-learning phase. Half of the participants who performed the dual-task in the learning phase continued to perform the dual-task (*consistent context*), and the other half performed only the primary visuomotor adaptation task (*inconsistent context*). We confirmed that both young (75% ± 3.4, t(14) = 12.34, p < 0.01, d = 3.3) and older (67%, ± 3.5, t(14) = 9.88, p < 0.01, d = 2.55) adults performed the RSVP task significantly above chance (33%) in the consistent context group. They were not different between the young and older adults, t(27) = 1.7, p = 0.1.

**Figure 4:**
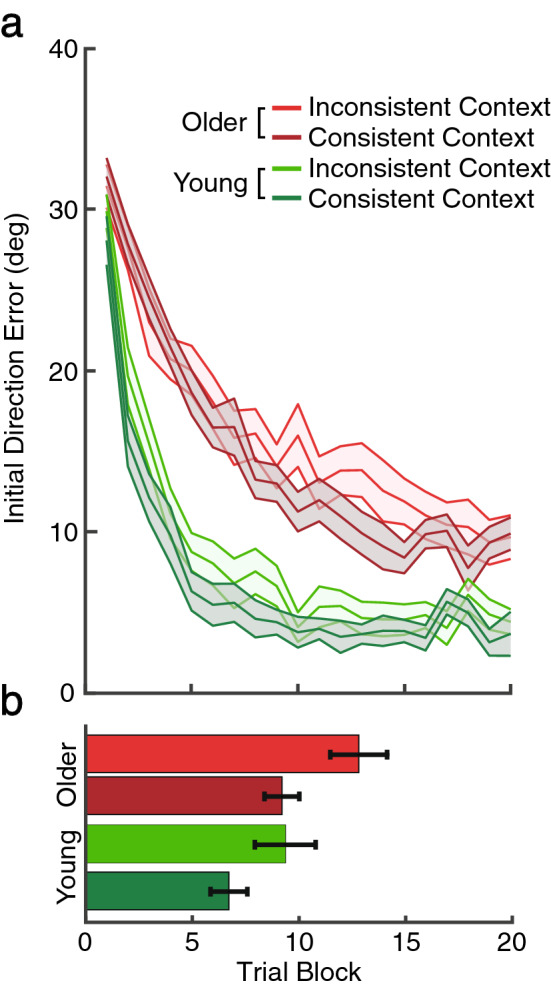
Visuomotor adaptation performance in the re-learning phase. We compare the re-learning performance of young (green) and older (red) adults from the dual-task groups in the learning phase. Within each age cohort, the inconsistent context group, who performed the secondary task in the learning but not in the re-learning phase, and the consistent context group, who continued to perform the secondary task, are indicated by the light and dark colors, respectively. (**a**) Initial direction error in the re-learning phase. (**b**) The number of trial blocks required to reach asymptotic initial direction error during the re-learning phase. Error bars indicate S.E.

A remaining key question is whether re-learning of the visuomotor adaptation is affected by a change in attentional context between the learning and re-learning phase. Figure 4a shows the mean initial direction error for each group in the re-learning phase. It appears that the consistent context group (darker line) reduces initial direction errors more rapidly than the inconsistent context group (lighter line) in both young (green) and older adults (red). To quantify re-learning performance in the consistent and inconsistent groups, we analyzed how quickly they reached their asymptotic performance measured in the learning phase.

Figure 4b represents when each group reached their previous asymptotic level, with lower values indicating faster re-learning or a stronger savings effect. Overall, young adults required fewer trials to reach their asymptotic error compared to older adult participants. Critically, participants required fewer trials to reach asymptotic error in the consistent than in the inconsistent context groups, and this effect was observed for both age groups. A two-way ANOVA of age (young vs. older) and context (consistent vs. inconsistent) confirmed these observations as both the main factors of age, F(1, 54) = 6.76, p = 0.01, η^2^ = 0.11, MSE = 18.83, and context, F(1, 54) = 7.49, p < 0.01, η^2^ = 0.11, were significant. The interaction of the two main factors was not significant (F < 1). This result suggests that, for both young and older adult participants, re-learning of visuomotor adaptation was superior when it was performed in the context that matched initial training.

**Figure 5:**
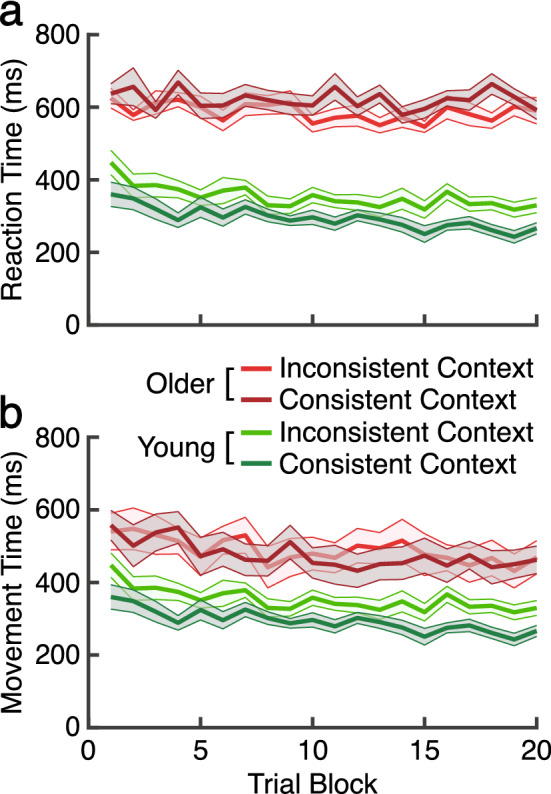
Reaction time and movement time during re-learning for the single and dual-task groups in young and older adults. Young and older groups are represented in green and red, respectively. Within each age group, the single-task and dual-task groups are represented by the dark and light-colored lines, respectively. Ribbons indicate SE. (**a**) Reaction time. (**b**) Movement time.

To confirm that these differences were not driven by different reaction time (RT) or movement time (MT), we also analyzed RT and MT to determine whether participants used a different reaching strategy in the context and inconsistent context conditions shown in Fig. 5. Specifically, while young adults were faster to initiate movement compared to older adults, mean, both age groups commonly reduced RT across trial blocks (Fig. 5a). The *age* × *secondary task* × *trial block* ANOVA confirmed these observation: significant main effects of age, F(1, 83) = 66.57, p < 0.001, η^2^ = 0.43, MSE = 0.28, and trial block, F(16.82, 1396.47) = 9.03, p < 0.001, η^2^ = 0.09, MSE = 0.02. However, the main effect of the secondary task was not significant (F < 1). Simple main effect analyses of each age group also confirmed that the mean RT is not different between the consistent and inconsistent groups for either the young, F(1, 40) = 1.95, p = 0.17, or older adult participants, F(1, 43) = 3.75, p = 0.06, η^2^ = 0.08, MSE = 0.28.

Similar pattern was also observed in MT (Fig. 5b). We observed significant main effects of age, F(1, 83) = 63.9, p < 0.001, η^2^ = 0.43, MSE = 0.47 and block, F(16.84, 1398.08) = 6.9, p < 0.001, η^2^ = 0.07, MSE = 0.03. However, the effect of secondary task was not significant (F < 1). Simple main effect analysis of each group also showed that MT was not affected by the secondary task for both young (F < 1) and older adults, F(1, 43) = 1.53, p = 0.22, η^2^ = 0.03, MSE = 0.71. These observations in RT and MT are consistent with our previous studies that have used a similar dual-task paradigm, demonstrating no sign of participants utilizing a different reaching strategy in each group^20–23,36^.

## Discussion

In many daily activities, visuomotor skills are learned and applied in complex environments, where multiple stimuli compete for limited attentional resources. While learning to drive a new car, for example, the magnitude of vehicle movement in response to the amount of wheel turn and accelerator depression varies across vehicles. Thus, the driver must learn the new mapping between his or her actions and the resulting vehicle movement. Concurrently, they also must divide their limited attentional resources between maneuvering the car and many other tasks, such as looking in the mirror, using turn signals, and avoiding pedestrians.

To simulate such a real-world scenario with full of multisensory distractors in order to obtain a better understanding of how age-related cognitive decline interact with motor learning processes, we used a novel dual-task paradigm^20,22,36^ that combines a visuomotor adaptation learning task with a rapid serial visual presentation (RSVP) discrimination task (Fig. 1).

Consistent with prior work and common expectations, we observed that older adults overall showed a deterioration in performance on both the primary visuomotor and secondary cognitive tasks compared to younger adults^15,32,37–41^. Beyond this age-related deterioration in overall performance, however, we discovered that simultaneously coping with additional cognitive loads under dual-task conditions affected the relative pattern of sensorimotor learning differently for young and older adults. In younger adults, dividing attentional resources with a secondary cognitive task specifically impaired the early stage of adaptation learning that requires cognitive resources. In older adults, however, dual-task conditions did not impair this early stage but instead enhanced visuomotor adaptation in the late stage for older adults. Thus, and in accordance with the compensatory hypothesis, older adult performers could obtain better adaptation learning outcomes by shifting to a more automatic mode of control, allowing the utilization of unconscious, fast, and reflexive learning processes.

In general, older adults involve more widespread brain regions during the performance of motor control tasks than young adults, particularly the prefrontal cortex and basal ganglia networks. Age-related neural degradation is compensated by greater activation and recruitment of brain areas mediating top-down working memory processes in order to maintain task performance. Unfortunately, these same regions are the most vulnerable to age-related effects, resulting in an imbalance of “supply and demand”^42^. Thus, at the high levels of demand typically seen under dual-task conditions, these compensatory mechanisms will no longer be effective, and older adults would begin to display disproportionately greater decrements in task performance compared to young adults. In visuomotor adaptation, however, we further suggest that when older adults are engaged in the secondary cognitive task that additionally depletes cognitive resources, they shift their reliance from cognitive control to implicit learning processes, which in turn more effectively automatizes motor learning. Thus, the implicit learning processes supporting adaptation learning may, in essence, provide a secondary compensation strategy that can be evoked when the primary attentional compensatory strategies are no longer sufficient to maintain performance.

In addition to exploring how allocating attentional resources to a concurrent secondary task interacts with age-dependent visuomotor adaptation, we also examined whether the success of motor memory retrieval depends on whether participants consistently perform the secondary task during later re-learning stages as well as initial learning, and whether this dependence is modulated by aging. In prior studies with young adults using a similar dual-task paradigm, Song and Bédard^20^ first reported that when participants were tested during a later re-learning phase, a motor skill initially learned under dual-task conditions was remembered only if a similar secondary task was also present during re-learning; when participants were instead tested without the secondary task, their performance reverted to untrained levels as though the motor task had not been learned in the first place. Here, we replicated this pattern of results in both young and old adults. This diverging result again suggests that while the secondary task does not interrupt initial motor memory formation, the dual-task context does act as a vital context for learning. Im et al.^22^ further showed that this dual-task context forms a long-term internal context affecting visuomotor performance on the following day.

Taken together, our work has demonstrated unexpected similarities and differences in the ways in which concurrent cognitive load modulates visuomotor learning in young and older adults. These findings provide new insights into the mechanisms mediating current dual-task performance as well as the development of long-term motor skills. It also highlights important practical implications for designing aging-appropriate motor learning programs to be more efficient and generalizable to dynamic real-world settings. We believe that characterization of this cross-integration mechanism will help develop a theoretical framework that describes how visual input interacts with attention and memory to generate motor actions. Furthermore, emphasizing the interactions between motor and cognitive processes rather than simply focusing on the distinctions will, in turn, contribute to advancing the interdisciplinary fields of cognitive science, neuroscience, and biomedical engineering—all of which have studied cognitive processes and motor control separately.

## Methods

### Participants

Thirty-six undergraduate students (20.1 years [SD = 2.1 years], 15 females) participated in the experiment in exchange for course credit, and 45 older adult participants (72.0 years [SD = 7.1 years], 12 females, 15.69 years of education [SD = 0.33 years]), recruited from outside the university community, participated in the experiment. Older adults were administered the Mini-Mental State Examination (MMSE)^43^ and the Repeatable Battery for the Assessment of Neuropsychological Status (RBANS)^44^ for screening of their global cognitive status. All participants were self-reported right-hand dominant with normal or corrected-to-normal vision. The mean psychometric data for the older adults in each condition are presented in Table 2.

**Table 2:** Mean age, years of education, Mini-Mental State Examination (MMSE)^43^, and repeatable battery for the Assessment of Neuropsychological Status (RBANS)^44^ for each of the three older adult groups.

Group	Single-task (n = 15)	Dual-task	
		Consistent context (n = 15)	Inconsistent context (n = 15)
Age	71.67 (1.61)	69.93 (1.82)	71.33 (2.02)
Years of education	16.13 (0.59)	15.07 (0.63)	15.87 (0.47)
MMSE (score)	29.13 (0.23)	28.47 (0.34)	28.73 (0.27)
RBANS total (standard score)	105.13 (3.79)	100.08 (2.76)	107.07 (3.47)

Numbers in the parentheses indicate S.E.

All participants had normal color vision, normal or corrected-to-normal vision, and were naïve to the goal of the experiment. The number of participants per group was determined on the basis of prior studies that utilized a similar dual-task paradigm and experimental design^20–23,36^. Reliable effect sizes were observed in these studies (*η*^2^ > 0.26). According to Cohen^45^, effect sizes of 0.02, 0.13, and 0.26 are considered small, medium, and large effect sizes, respectively. The sample size is also within the range of sample sizes from previous visuomotor adaptation studies^46,47^. The experimental protocol was approved by the Brown University Institutional Review Board (IRB) in accordance with the Code of Ethics of the World Medical Association (Declaration of Helsinki) for experiments involving humans. All research was performed in accordance with the approved IRB guidelines. The informed consent was obtained from all the participants.

### Apparatus

Participants sat in a dimly lit room roughly approximately 60 cm from an Apple iMac computer with a 21-inch screen with a refresh rate of 60 Hz and the native resolution of 1920 × 1080 pixels. Participants used a stylus pen to perform a goal-directed reaching task with their right hand. Movement of the stylus controlled a corresponding cursor on the screen (diameter 0.5 cm). The tip of the stylus rested on a touchpad (Magic Touch; Keytec, Garland TX) that lay flat on a table and aligned with each participant's midline and the center of the computer screen. Stimulus presentation and recording of cursor position were controlled by custom software written in MATLAB (version 2016b; Mathworks, Natick, MA) and functions from Psychtoolbox (version 3)^48,49^.

### Tasks

#### Primary visuomotor adaptation task (VM)

In the primary VM task (Fig. 1a), a trial started when the cursor was positioned in the starting position, and this triggered the appearance of the reach target in the visuomotor task, as well as the visual stream in the secondary task. All participants were instructed to move a cursor from a starting position (annulus with a diameter of 1°, corresponding to 1 cm) in the center of the screen towards a visible reach target (white dot with 1 cm in diameter on a black background) located 7.5 cm away at 3, 6, 9, and 12 o’clock in relation to the starting base. Each target location was presented in a pseudorandomized order within blocks of four trials. A trial started when the cursor was positioned in the starting position, and this triggered the appearance of the reach target in the visuomotor task, as well as the visual stream in the secondary task. There were two types of experimental trials. In the no-rotation trials, the cursor position followed the stylus movement. In the rotation trials, the cursor was rotated 45° counterclockwise (CCW). The instructions emphasized that participants should move quickly and accurately toward the target in a straight line, discouraging corrective movements, and then return to the starting position immediately after reaching the target. The target remained visible for the entire duration of the trial (i.e., 1500 ms aligned the duration of the secondary task), including both the outward and inward movements. Outside trials, the unrotated cursor was visible to guide participants back to the starting location. There were the four experimental phases: (1) baseline (40 no-rotation trials with four target locations), (2) learning (160 rotation trials with four target locations), (3) washout (80 no-rotation trials with four target locations), and (4) re-learning (80 no-rotation trials with four target locations). The baseline phase was designed to measure for inherent bias in the reach movement toward each direction. In the training phase, visuomotor adaptation was trained on all four targets. Participants received continuous feedback to all targets, and cursor movement was rotated either 45° CCW from stylus movement. In the washout phase, participants de-adapted, moving again without rotation towards all four learning targets. Finally, in the re-learning phase, participants were exposed to the same rotation (CCW) they had experienced in the learning phase.

### Secondary rapid serial visual presentation (RSVP) task

Figure 1b illustrates the schematic outline of the RSVP task. A sequence of five T shapes (0.5° × 1°) was generated from pseudorandom permutations of letter orientation (inverted or upright) and color (red, white, green, blue, or yellow). The sequence was presented directly above the starting position, and each T in the sequence appeared every 300 ms, remaining visible for only 150 ms (for a total of 1500 ms). Participants were required to report the total number of green targets (regardless of orientation) at the end of each trial using their left hand. The number of target Ts in the sequence varied between one and three along with a uniform distribution—as a result, 33% accuracy represents chance performance. At the end of each trial, participants reported the number of targets observed (one, two, or three) by pressing a key on a computer keyboard with the left hand. To control for the effect of divided attention on reaching, a control task was devised in which participants received instructions to ignore the Ts and simply press the key corresponding to a visual cue (e.g., “Press button 1”). Participants received feedback following their button response, with correct and incorrect responses indicated by a soft beep and loud buzz, respectively. Note that Ts appeared on every trial for all four VM task phases, so the visual stimuli were the same across all participants. Eye movements were not constrained throughout the experiment, as previous studies found no evidence that eye movements affected performance in the dual-task paradigm^20^.

### Procedure

All participants performed the visuomotor adaptation task (Fig. 1a), but the performance of the RSVP task (Fig. 1b) was dependent on group assignment (RSVP vs. none) and the experimental phase, as indicated in Table 1. To summarize, we randomly assigned young and older participants separately to one of three groups, labeled according to whether participants performed the RSVP task during the learning (single vs. dual-task group) and then among the dual-task group, to whether they consistently performed the dual-task in the re-learning phase (consistent context vs. inconsistent context). For young adult groups, 14 participants were assigned to each group, and 15 participants were assigned to each older adult group.

Each trial began after the cursor had been stationed in the starting position for 1 s, which then triggered the appearance of the reach target as well as the commencement of the visual stream in the secondary task. A trial began after the cursor had been stationed in the starting position for one second, which then triggers the appearance of the reach target as well as the commencement of the visual stream in the secondary task. All participants familiarized themselves with the experimental procedure by completing separate practice blocks of the reaching (20 no-rotation trials) and RSVP task (20 trials), as well as a third practice block of the reaching task with the concurrent secondary task.

### Data analysis

Data analysis procedures followed those outlined in our previous studies^20–23,36^. For the visuomotor task, we filtered the *x* and *y* coordinates of the cursor displacements with a low-pass Butterworth filter using a 10 Hz cutoff and then calculated the cursor trajectory by taking the square root of the sum of squared *x* and *y* coordinates at each time point. We differentiated the position of the cursor to yield tangential velocity and determined the onset and end of the movement when the cursor reached 5% of peak velocity. Reaction time (RT) is the time elapsed from target appearance to movement onset and the movement time (MT) is the time elapsed between movement onset and movement end. Initial direction error was determined by calculating the angle between the line that joined the starting position to the target with the line that joined the position of the cursor at movement onset to the position of the cursor at peak velocity. Given that participants were explicitly instructed to reach the target in a straight trajectory and without corrective movements, initial direction error reflects both the accuracy and efficiency of reach movements.

To conduct the statistical analysis, each dependent variable (i.e., initial direction error, RT, and MT) was averaged across four successive trials to create 40 blocks of four trials in learning and 20 blocks of four trials in washout and re-learning. Thus, each trial block included a performance from four target locations. We used mixed-effect ANOVAs with *age* (young vs. older adult) and *secondary task* (single vs. dual) as a between-subjects factor, *trial block* as repeated measures. A Mauchly's test of sphericity revealed that the assumption of sphericity was violated for the repeated-means factor of a trial block. Greenhouse–Geisser corrections were applied to subsequent ANOVAs involving this factor to reduce the inflation of Type I error.

We also conducted an additional analysis to calculate asymptotic initial direction error (see also Im et al.^21^). We first estimated the elbow point of each participant’s learning curve by fitting the curve to the following exponential function:

$$y=a{e}^{b}+c,$$

where *a* is the y-intercept, *b* is the rate of learning and *c* is the level of the low asymptote. Using the fitted parameter *c*, reflecting the low asymptote of the learning curve. We then calculated the trial block in which the participant reached their individual learning asymptote by finding the point in which the learning curve intersects with the value of the *c* parameter. For each participant, the asymptotic initial reach error is the mean reach error from the start of this intersection point to the end of the learning phase. The mean asymptotic initial direction error is illustrated in Fig. 2a.
